# LncRNA RARA-AS1 could serve as a novel prognostic biomarker in pan-cancer and promote proliferation and migration in glioblastoma

**DOI:** 10.1038/s41598-023-44677-4

**Published:** 2023-10-13

**Authors:** Yue Huang, Song Deng, Qiaoji Jiang, Jinlong Shi

**Affiliations:** 1 Department of Neurosurgery, Affiliated Hospital of Nantong University, Medical School of Nantong University, No. 20 West Temple Road, Nantong, 226001 Jiangsu China; 2 Department of Neurosurgery, Affiliated Yancheng Clinical College of Xuzhou Medical University, Yancheng, 224000 Jiangsu China

**Keywords:** Long non-coding RNAs, Data mining, Prognostic markers, Oncogenes

## Abstract

Long non-coding RNAs (lncRNAs) have emerged as crucial regulators of cancer progression and are potential biomarkers for diagnosis and treatment. This study investigates the role of RARA Antisense RNA 1 (RARA-AS1) in cancer and its implications for diagnosis and treatment. Various bioinformatics tools were conducted to analyze the expression patterns, immune-related functions, methylation, and gene expression correlations of RARA-AS1, mainly including the comparisons of different subgroups and correlation analyses between RARA-AS1 expression and other factors. Furthermore, we used short hairpin RNA to perform knockdown experiments, investigating the effects of RARA-AS1 on cell proliferation, invasion, and migration in glioblastoma. Our results revealed that RARA-AS1 has distinct expression patterns in different cancers and exhibits notable correlation with prognosis. Additionally, RARA-AS1 is highly correlated with certain immune checkpoints and mismatch repair genes, indicating its potential role in immune infiltration and related immunotherapy. Further analysis identified potential effective drugs for RARA-AS1 and demonstrated its potential RNA binding protein (RBP) mechanism in glioblastoma. Besides, a series of functional experiments indicated inhibiting RARA-AS1 could decrease cell proliferation, invasion, and migration of glioblastoma cell lines. Finally, RARA-AS1 could act as an independent prognostic factor for glioblastoma patients and may serve as a promising therapeutic target. All in all, Our study provides a comprehensive understanding of the functions and implications of RARA-AS1 in pan-cancer, highlighting it as a promising biomarker for survival. It is also an independent risk factor affecting prognosis in glioblastoma and an important factor affecting proliferation and migration in glioblastoma, setting the stage for further mechanistic investigations.

## Introduction

Cancer cases are predicted to exceed 1,958,310 in 2023 and deaths from cancer are predicted to reach 609,820, which is 1670 deaths per day^1^. Currently, cancer treatment includes conventional drugs and surgery, as well as emerging targeted inhibitors, photodynamic therapy^2^, and immunotherapy^3^. These methods have been successful in clinical practice and are constantly evolving^4^. However, due to limited efficacy and recurrence limitations, the prognosis and tolerance of cancer patients are still unsatisfactory. Fortunately, transcriptomics, as a rapidly developing technology, has become a critical tool in cancer’s research^5^. Transcriptomics also plays an increasingly important role in personalized cancer therapy^6^. For certain specific types of cancer, by detecting their transcriptome levels, it is possible to predict a patient's response to different treatment regimens and guide doctors in selecting the optimal treatment strategy. In addition, by using transcriptomic techniques, new treatment targets and drugs can also be discovered, promoting further development in the field of cancer treatment. Overall, transcriptomics has become an indispensable technology in the field of cancer research and treatment, providing more comprehensive information for early detection and diagnosis of cancer, personalized precision treatment, and discovery of new drug targets.

Neuroglioblastoma is a malignant tumor that originates in the central nervous system. Among them, glioblastoma accounts for more than 50%^7^. Despite some progress in the treatment of glioblastomas^8^, its prognosis remains poor, with some reasons including treatment resistance, recurrence, and metastasis^9^. Surgical resection is currently the main method for treating glioblastomas. However, due to special reasons such as the location of the tumor and extramedullary growth, surgical resection is often difficult and cannot be completely cleared. In recent years, new treatment methods have begun to be applied in the field of glioblastomas^10^. Immunotherapy, which boosts the immune system of patients to fight cancer cells, has become an important treatment for tumors^11^. At the same time, some tumor molecular markers have shown increasingly positive roles in the field of glioblastomas^12–14^. These biomarkers can be used to evaluate patients' prognosis and treatment response, providing important guidance for personalized precision medicine. In the future, personalized precision medicine based on tumor molecular markers is expected to become a new choice for glioblastoma treatment. Therefore, identifying key biomarkers to accurately estimate the survival outcomes of glioblastoma patients has become an urgent task.

**Figure 1:**
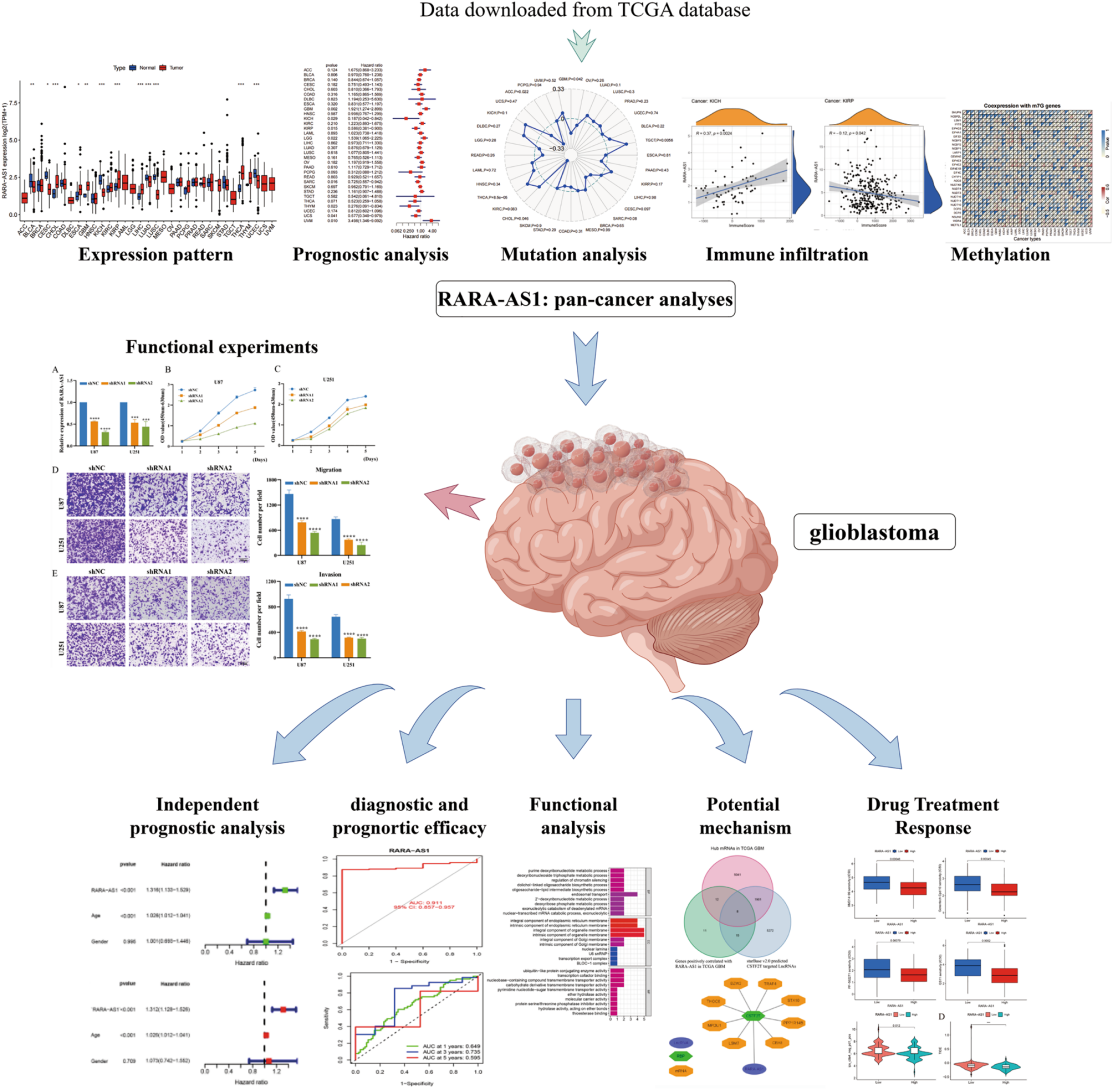
Workflow of this whole study.

LncRNA RARA Antisense RNA 1 (RARA-AS1) is located on chromosome 17q21.2 and has been reported to be differentially expressed in septic patients, and may be a potential target for this disease^15^. In order to better understand RARA-AS1 and its relationship to various cancer outcomes, it is necessary to examine this gene in more detail. Therefore, we mainly used large-scale genomic data from the Cancer Genome Atlas (TCGA) to perform pan-cancer analysis of RARA-AS1, including its expression patterns by comparisons between tumor and normal samples, as well as its relationship with survival via univariate cox regression analyses. And the correlation analyses were conducted to investigate the correlation between RARA-AS1 and mutation, immune infiltration, and methylation. In addition, we particularly emphasized the role of RARA-AS1 in glioblastoma, mainly including independent prognostic analyses, diagnostic and prognostic efficacy, functional analysis, potential mechanism, and drug treatment response. In addition, the functional experiments were performed by us identify the biology behavior of RARA-AS1, consisting of CCK8, migration and invasion analyses. The workflow was shown in Fig. 1. Our study clarifies RARA-AS1’s role in glioblastoma biology behavior, and provides new insights into RARA-AS1’s potential as a pan-cancer biomarker and therapeutic target.

## Materials and methods

### Differential expression analysis

Data on gene expression, clinical characteristics, and phenotypes were downloaded from TCGA database (https://portal.gdc.cancer.gov/), by selecting the corresponding categories for the different tumors, including “transcriptome profiling” and “clinical”, respectively. And gene ID conversion was done based on the human reference genome file with version number GRCh38.p13. The gene expression data was normalized using log2(TPM + 1). Using R package “limma”, the gene expression data of RARA-AS1 for 33 types of tumors in the TCGA database was extracted and analyzed for differential expression between tumor tissue and normal tissue samples using Wilcoxon rank-sum test for each cancer type. Statistical significance was considered to exist when was p < 0.05, and gene expression levels were visualized using box plots. Further analysis of RARA-AS1 expression in normal tissues was performed using GTEx database, with expression normalization also using log2(TPM + 1), and this analysis was performed using the sangerbox website (http://past20.sangerbox.com/). R software (version 4.2.2; http://www.R-project.org) was used to process and analyze data, and the ggpubr package was used to generate box plots.

### Pan-cancer prognosis analysis

Since parametric tests are more potent than non-parametric tests, we chose COX regression analyses to complete the survival analyses. RARA-AS1 was examined using univariate Cox regression models for overall survival (OS), disease-specific survival (DSS), Disease Free Interval (DFI) and progression-free interval (PFI). In accordance with the median expression of RARA-AS1, samples were categorized as high-expression or low-expression. The “survival” package was used to conduct the analyses and “forestplot” was applied to visualize the results. Statistical significance was determined by a p-value less than 0.05.

### The correlation between the expression of RARA-AS1 and TMB, MSI, and NEO

On the basis of autosomal microsatellite tracts determined by MISA (http://pgrc.ipk-gatersleben.de/misa/misa), tumors were labeled as microsatellite (MSI)^16^ if more than two markers out of five showed MSI. The tumor mutation burden (TMB) was also calculated using the TCGA database by comparing gene mutations within tumor tissues to the corresponding gene mutations within adjacent normal tissues^17^. Additionally, we used the TCGA expression dataset to detect Neoantigen (TNB) without changing the original settings^18^. All these analyses were performed using online Sangerbox tools. The correlation method was pearson with a threshold p-value of 0.05.

### The correlation between RARA-AS1 and immune checkpoint molecules as well as the tumor microenvironment

RARA-AS1 and the immune microenvironment are evaluated by the ESTIMATE algorithm, including Immune, ESTIMATE, and Stromal scores. The concept of ESTIMATE is used to estimate how much immune cells and stromal cells infiltrate tumor tissue^19^. Immune and stromal scores were calculated using the “ESTIMATE” and “limma” packages in R for each case's tumor tissue. Putative correlations between RARA-AS1 expression and immune scores in different tumors were then evaluated. Furthermore, the CIBERSORT method is based on the principle of linear support vector regression for inverse convolution analysis. Then, in each case, the CIBERSORT algorithm in R was employed to determine the abundance of immune cell infiltration^20^, and its correlation with RARA-AS1 expression in different tumors was further analyzed. Correlation graphs were generated using the R “ggplot2,” “ggpubr,” and “ggExtra” packages. Above mentioned analyses were based on spearman and statistical significance was defined as a p-value less than 0.05.

Additionally, we correlated the expression level of RARA-AS1 in TCGA with mismatch repair proteins and immune checkpoint molecules using the online Sangerbox tool. The method used for this analysis was Pearson, and a p-value of less than 0.05 was considered significant. Moreover, the mutation status of RARA-AS1 was downloaded from the cBioPortal website (http://www.cbioportal.org/), providing clear observations of the gene's mutation types and corresponding mutation frequencies in different tumors.

### The correlation between RARA-AS1 and methylation

A collection of representative m6A-related genes, m7G-related genes, and methyltransferase genes were analyzed for the Pearson correlation with RARA-AS1 in the 33 types of tumors from the TCGA database, with the results presented in the form of a heatmap. The “limma” and “reshape2” packages in R were mainly used for this analysis and the correlation analyses were carried out by function “cor.test”, method “Pearson”, and threshold “p-value was less than 0.05”.

### Functional enrichment and RBP mechanism prediction of RARA-AS1

To explore the possible biological functions and pathways involved in RARA-AS1, differential analysis was conducted using high and low expression groups of RARA-AS1, followed by gene ontology (GO) and Kyoto Encyclopedia of Genes and Genomes (KEGG) pathway enrichment analysis^21^. Gene Set Enrichment Analysis (GSEA) was also performed^22^. In all three analyses above, a p-value of less than 0.05 was considered significant data. In addition, GO analyses show the top 10 of each category, KEGG analyses show all meaningful results, and GSEA analyses show the top 5 meaningful entries. In addition, to reveal the potential regulatory mechanisms of RARA-AS1 in glioblastoma, several RARA-AS1/RBP/mRNA networks were proposed. Using StarBase v2.0, RBPs targeted by RARA-AS1 were predicted with a threshold of high stringency (≥ 3), and pan-Cancer analysis included more than 10 types of cancer. Once potential RBPs had been identified, StarBase v2.0 was used again to predict their mRNA targets, with a strict threshold (≥ 5) and pan-Cancer analysis included more than 10 types of cancer. The hub mRNAs in TCGA had to have a p-value less than 0.05, |log2FC| ≥ 1, and FDR < 0.05. A meaningful positive correlation with RARA-AS1 was the last condition. The intersection of the genes filtered by the different conditions above is taken using a Wayne diagram, and we get the gene we need. The complete RARA-AS1/RBP/mRNA axis was then visualised using cytoscape 3.6.1.

### RARA-AS1-related drug prediction

To further explore the relationship between the expression of RARA-AS1 and drug sensitivity and predict the response to drug treatment in high and low expression groups of RARA-AS1, we performed related analysis using the “pRRophetic” package. The IC50 values of different groups were calculated, with lower IC50 values indicating potentially better efficacy when using a particular drug. Additionally, scores from the Tumor Immune Dysfunction and Exclusion (TIDE) database were used to predict whether glioblastoma patients might benefit from immunotherapy^23^. Patients with a higher TIDE score are more likely to be immune excluded, meaning immunotherapy is less likely to be beneficial. Scores from The Cancer Immunome Atlas (TCIA) database were used to predict patient responses to PD1 and CTLA4 targeted therapies^24^. The statistical method for comparison between groups was Wilcoxon rank-sum test and all analyses were considered statistically significant if the p-value was less than 0.05.

### Biological functional analysis of RARA-AS1

The U87 (Catalog No: CL-0238) and U251 (Catalog No: CL-0237) cell lines were purchased from Procell (Wuhan, China). They were cultured to 60–70% confluence and transfected with short hairpin RNA negative control (shNC), shRNA1, or shRNA2 using Lipofectamine 3000 (Thermo Fisher Scientific) and harvested 24–48 h later. For CCK-8 experiments, cells were counted 24–48 h after transfection, diluted to 3 × 10^4^ cells/mL, and seeded into a 96-well plate. Cells were incubated for 24, 48, 72, 96, or 120 h, with each time point repeated four times. Then, each well was added with 10 μL of CCK-8 reagent at the corresponding time point, which was offered by Donjindo, Kumamoto, Japan. Absorbance at 450 and 630 nm was measured after 2 h using a microplate reader. For transwell experiments, cell density was adjusted to 5 × 10^5^ cells/mL for cell migration assays and 8 × 10^5^ cells/mL for cell invasion assays. DMEM medium (500 μL) was added to a 24-well plate, and then the transwell inserts were added to the plate. In invasion experiments, the upper chamber was filled with Matrigel (Corning) mixed with DMEM medium at a 1:6 ratio. We incubated cells in the suspension for 48 h before fixing them with paraformaldehyde, staining them with crystal violet, and taking micrographs. Additionally, qRT-PCR experiments were performed using previously described methods^25^ to compare the relative expression levels between groups. The primer sequences used are listed below: RARA-AS1 (F 5′-CTCAAAGTTCCTCAGCCCTAATC-3′, R 5′-CCCTTGCTGGACAATTGAACC-3′) and 18S (F 5′-CGGCTACCACATCCAAGGAA-3′, R 5′-GCTGGAATTACCGCGGCT-3′). In the above experiment, the statistical method used between the different subgroups was ANOVA and Tukey's HSD test, and also a p-value of less than 0.05 was considered as a conditional difference.

### Ethics approval and consent to participate

The study was approved by the Institutional Research Ethics Committees of Affiliated Hospital of Nantong University.

## Results

### Expression of RARA-AS1 in pan-cancer

**Figure 2:**
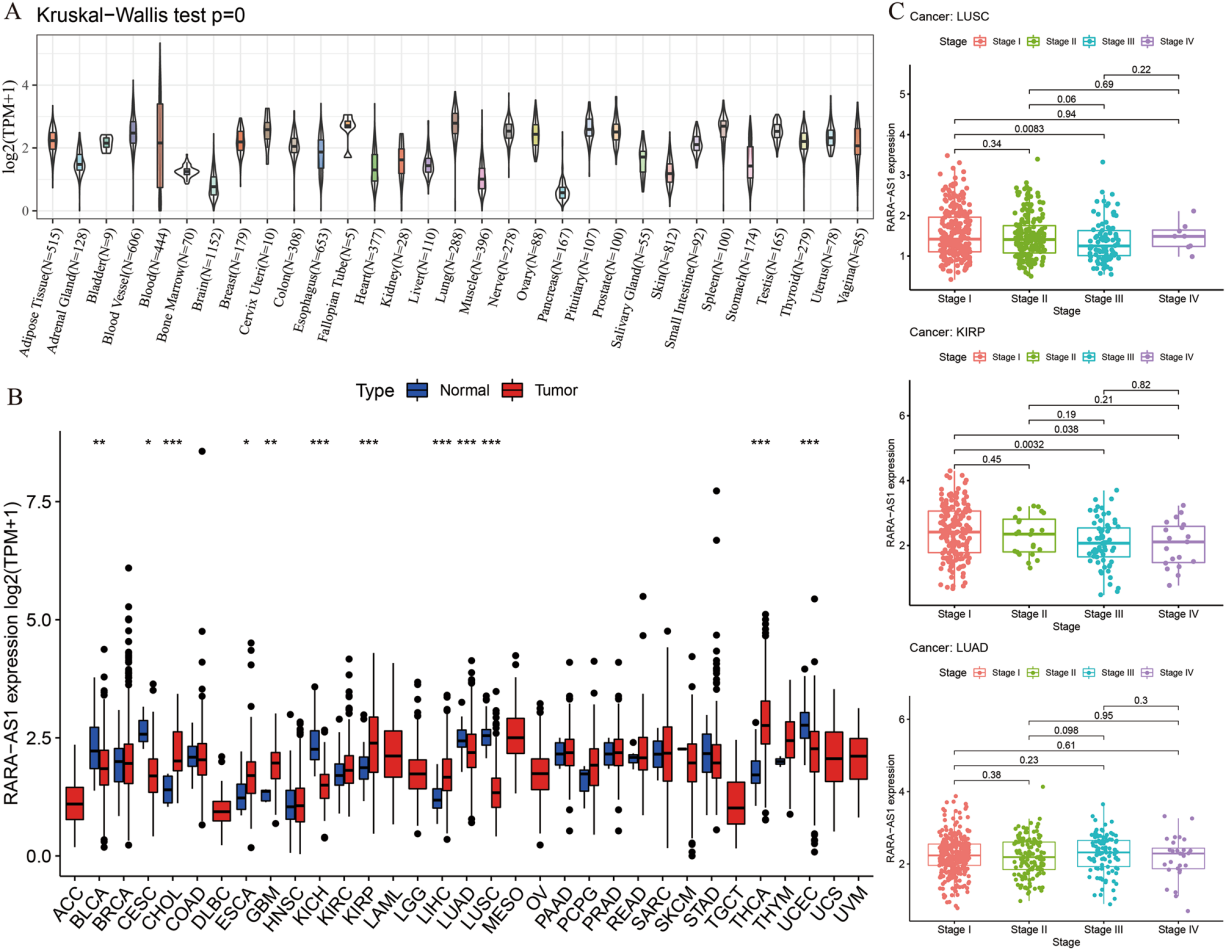
Expression level of RARA-AS1 in tumors and normal tissues. (**A**) RARA-AS1 expression in normal tissues; (**B**) RARA-AS1 expression in TCGA tumors and normal tissues; (**C**) RARA-AS1 expression in different stages in LUSC, KIRP, and LUAD. *p < 0.05, **p < 0.01, ***p < 0.001.

Using the GTEx database of normal tissues, our study showed that RARA-AS1 is highly expressed in cervix uteri, lung, spleen and pituitary, while its expression is relatively low in brain and pancreas (Fig. 2A). Further analysis of transcriptome data from 33 types of cancer in TCGA revealed differential expression of RARA-AS1 in 12 types of cancer. Notably, it is significantly downregulated in Bladder urothelial carcinoma (BLCA), Cervical squamous cell carcinoma and endocervical adenocarcinoma (CESC), Kidney chromophobe (KICH), Lung adenocarcinoma (LUAD), Lung squamous cell carcinoma (LUSC), and Uterine corpus endometrial carcinoma (UCEC), but upregulated in Cholangiocarcinoma (CHOL), Esophageal carcinoma (ESCA), Glioblastoma multiforme (GBM), Kidney renal papillary cell carcinoma (KIRP), Liver hepatocellular carcinoma (LIHC), and Thyroid carcinoma (THCA) (Fig. 2B). The expression of RARA-AS1 in LUSC, KIRP, and LUAD also showed a decreasing trend with advanced stage (Fig. 2C). Overall, RARA-AS1 exhibits diverse expression patterns in pan-cancer and has the potential to serve as a biomarker.

### Prognostic analysis of RARA-AS1 in pan-cancer

**Figure 3:**
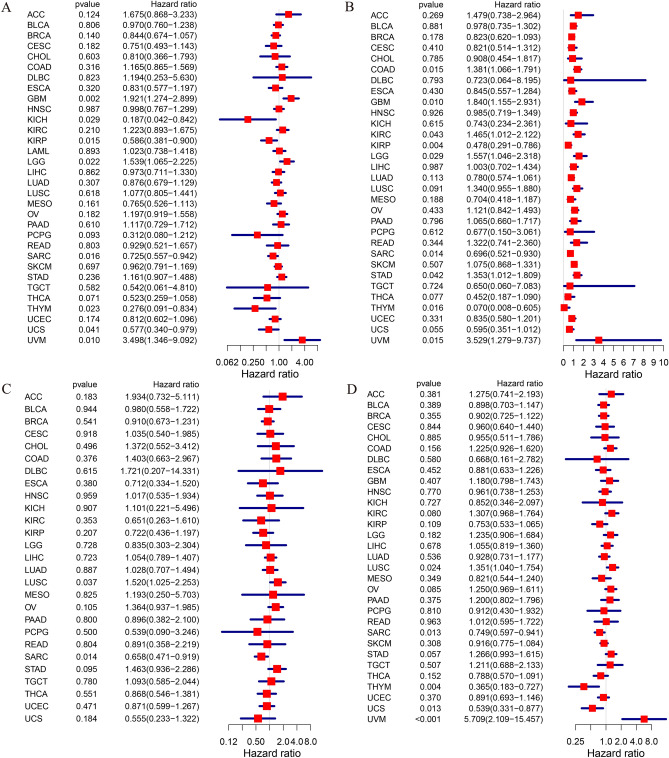
Univariate regression analyses of RARA-AS1 in pan-cancer. (**A**) Association between RARA-AS1 expression and OS; (**B**) Relationship between RARA-AS1 expression and DSS; (**C**) Association between RARA-AS1 expression and DFI; (**D**) Association between RARA-AS1 expression and PFI. p < 0.05 was considered to be significant.

Univariate Cox regression analysis of OS in pan-cancer patients shows that the expression of RARA-AS1 is significantly associated with poor survival outcomes in GBM, KICH, KIRP, Brain lower grade glioma (LGG), Sarcoma (SARC), Thymoma (THYM), Uterine carcinosarcoma (UCS), and Uveal melanoma (UVM). Specifically, high expression levels of RARA-AS1 are associated with poorer OS in GBM, LGG, and UVM, whereas the opposite trend is observed in KICH, KIRP, SARC, THYM, and UCS (Fig. 3A). Moreover, the results from DSS analysis indicate that high expression of RARA-AS1 is also associated with poorer survival in Colon adenocarcinoma (COAD), GBM, Kidney renal clear cell carcinoma (KIRC), LGG, Stomach adenocarcinoma (STAD), and UVM, while the opposite trend is observed in KIRP, SARC, and THYM (Fig. 3B). In addition, the results from DFI analysis show that high expression of RARA-AS1 is associated with poor DFI only in LUSC, whereas the opposite trend is observed in SARC (Fig. 3C). Furthermore, the results from PFI analysis demonstrate that high expression of RARA-AS1 is a risk factor for LUSC and UVM, but a protective factor for SARC, THYM, and UCS (Fig. 3D).

### Correlation analysis of RARA-AS1 with immune checkpoints and mutation

**Figure 4:**
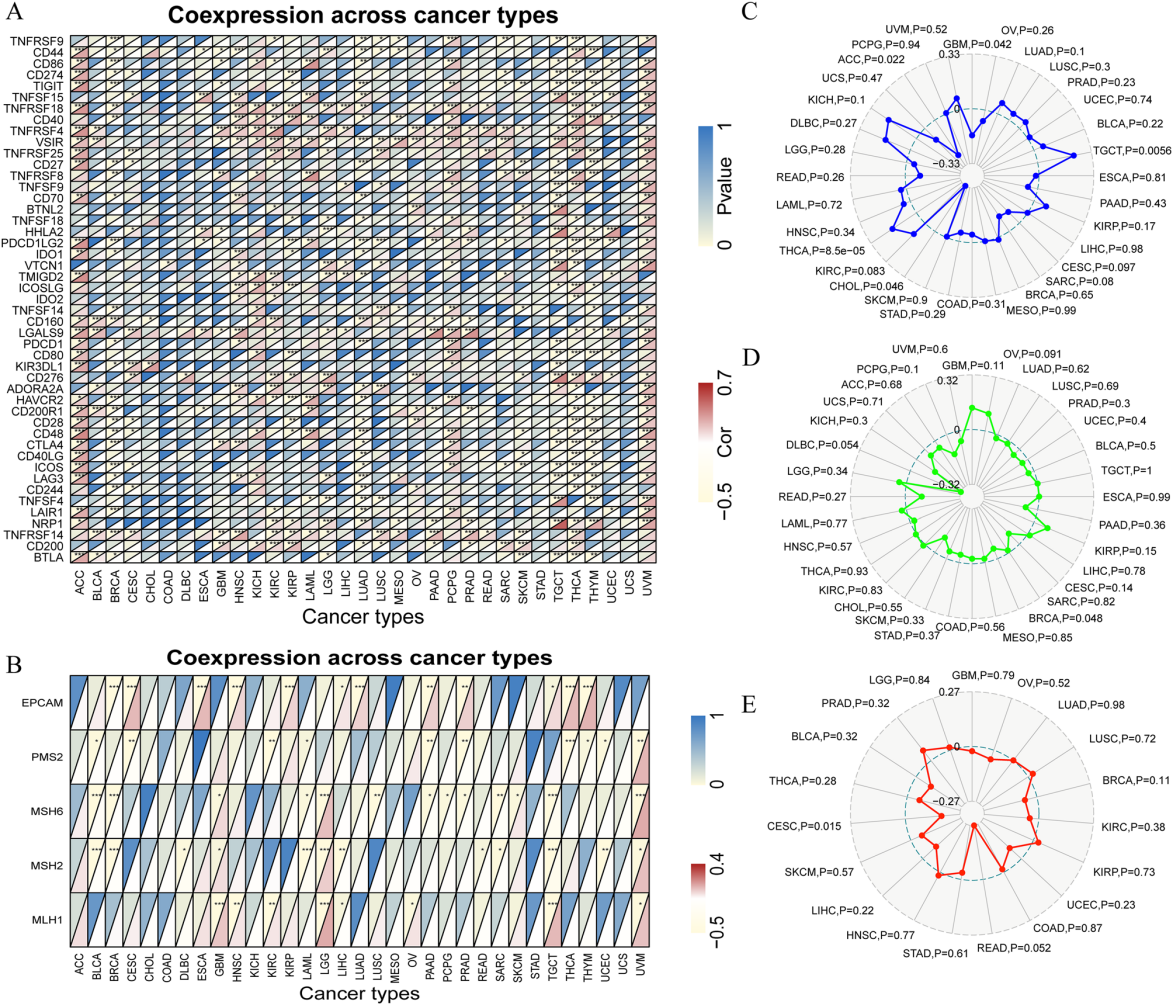
Mutation landscape and correlation analyses with RARA-AS1. (**A**) Correlation between RARA-AS1 expression and immune checkpoints; (**B**) Relationship between RARA-AS1 expression and MMR; (**C**) Association between RARA-AS1 expression and MSI; (**D**) Correlation between RARA-AS1 expression and TMB; (**E**) Association between RARA-AS1 expression and NEO. *p < 0.05, **p < 0.01, ***p < 0.001.

The correlation analysis between RARA-AS1 and immune checkpoints shows that most of the immune checkpoints are significantly associated with RARA-AS1 in Adrenocortical carcinoma (ACC) and UVM, with a correlation coefficient greater than 0.3 (Fig. 4A). Specifically, several immune checkpoints including TNFRSF18, TNFRSF4, VSIR, LGALS9, HAVCR2, and TNFRSF14 are significantly associated with RARA-AS1 in more than 10 types of cancer. Notably, VSIR, LGALS9, and TNFRSF14 are highly correlated with RARA-AS1 in 16 types of cancer, indicating their potential as therapeutic targets. Moreover, the correlation analysis between RARA-AS1 and MMR proteins reveals that at least three mismatch repair genes are significantly associated with RARA-AS1 in Breast invasive carcinoma (BRCA), GBM, LAML, LGG, LIHC, Pancreatic adenocarcinoma (PAAD), Testicular germ cell tumors (TGCT), and UVM (Fig. 4B).

To further investigate the relationship between RARA-AS1 and tumor heterogeneity, we conducted MSI, TMB, and NEO analyses. The pan-cancer analysis shows that RARA-AS1 is significantly correlated with MSI in five types of cancer, including GBM, CHOL, and ACC, which exhibit a negative correlation, whereas TGCT and THCA exhibit a positive correlation (Fig. 4C). Moreover, RARA-AS1 is negatively correlated with TMB only in BRCA (Fig. 4D). In addition, the NEO-related analysis reveals that RARA-AS1 is negatively correlated with NEO only in CESC, but not in other types of cancer (Fig. 4E). Furthermore, mutation analysis results indicate that RARA-AS1 has the highest mutation frequency in esophagogastric cancer, breast cancer, and adrenocortical carcinoma, with amplification as the main mutation form in the first two types of cancer and deep deletion as the main form in the latter (Supplementary Fig. 1).

### Correlation between RARA-AS1 and immune infiltration

**Figure 5:**
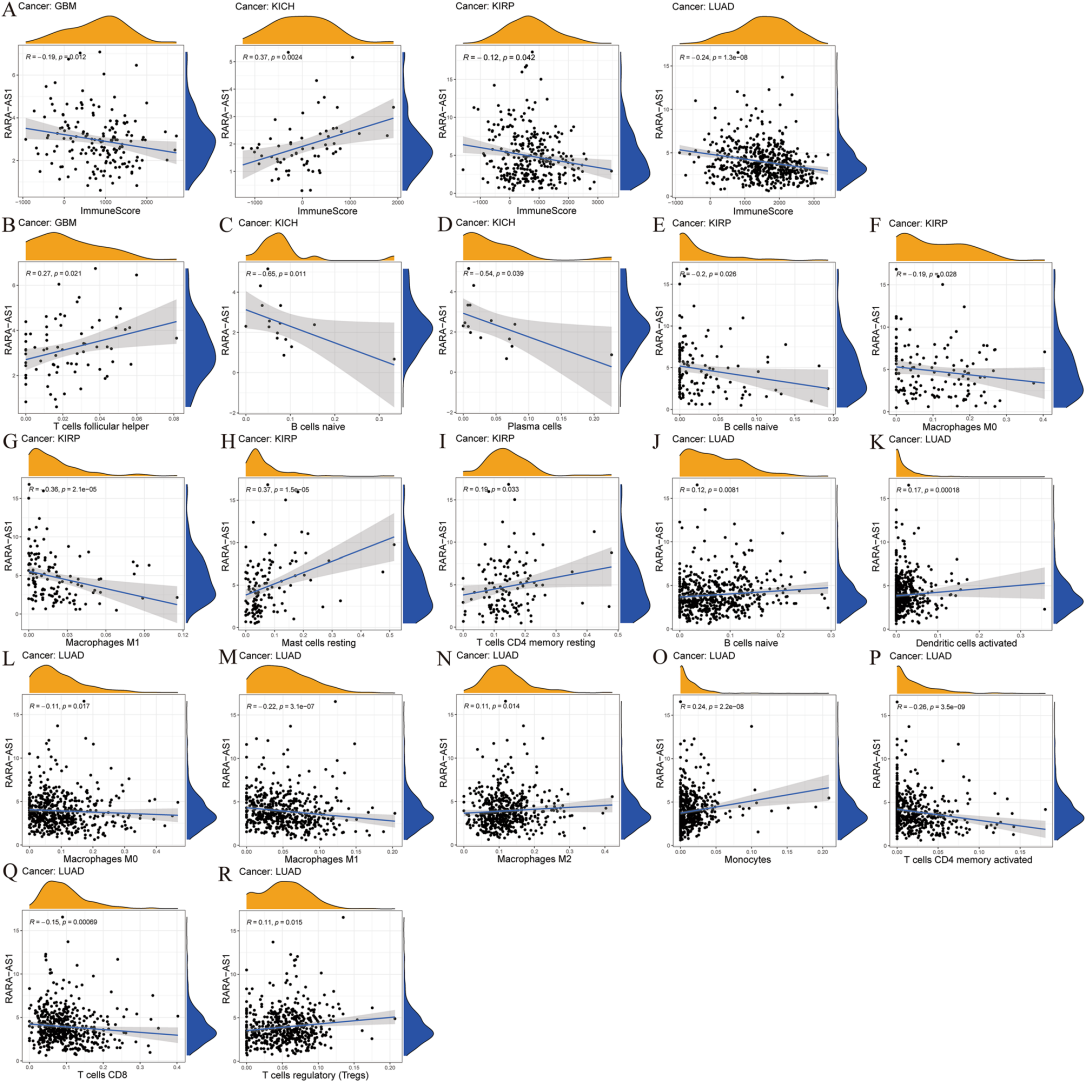
Correlation analysis between RARA-AS1 expression and immune infiltration. (**A**) Association (Estimate) between RARA-AS1 expression and Immune Score in patients with GBM, KICH, KIRP, and LUAD; Relationship (CIBERSORT) between RARA-AS1 expression and immune cells in patients with (**B**) GBM, (**C**,**D**) KICH, (**E**–**I**) KIRP, and (**J**–**R**) LUAD (p < 0.05).

We selected tumors with differential expression of RARA-AS1 in cancer tissues and adjacent normal tissues based on the results of ESTIMATE analysis (Fig. 5A). We found that the expression of RARA-AS1 was negatively correlated with Immunescore in GBM, KIRP, and LUAD, but positively correlated with KICH. Subsequently, we selected tumors consistent with the ESTIMATE analysis and displayed the results of CIBERSORT analysis significantly correlated with RARA-AS1 expression (Fig. 5B–R). T cells follicular helper cell in GBM, T cells CD4 memory resting and Mast cells resting in KIRP, B cells naïve, Macrophages M0, Macrophages M2, Monocytes, and T cells regulatory (Tregs) in LUAD were positively correlated with RARA-AS1, whereas all others were negatively correlated. Notably, B cells naive, Plasma cells in KICH were significantly negatively correlated with RARA-AS1, with a correlation coefficient less than 0.5. These analyses are critical to our understanding of the impact of RARA-AS1 on immune infiltration and related immunotherapy.

### Correlation between RARA-AS1 and methylation

**Figure 6:**
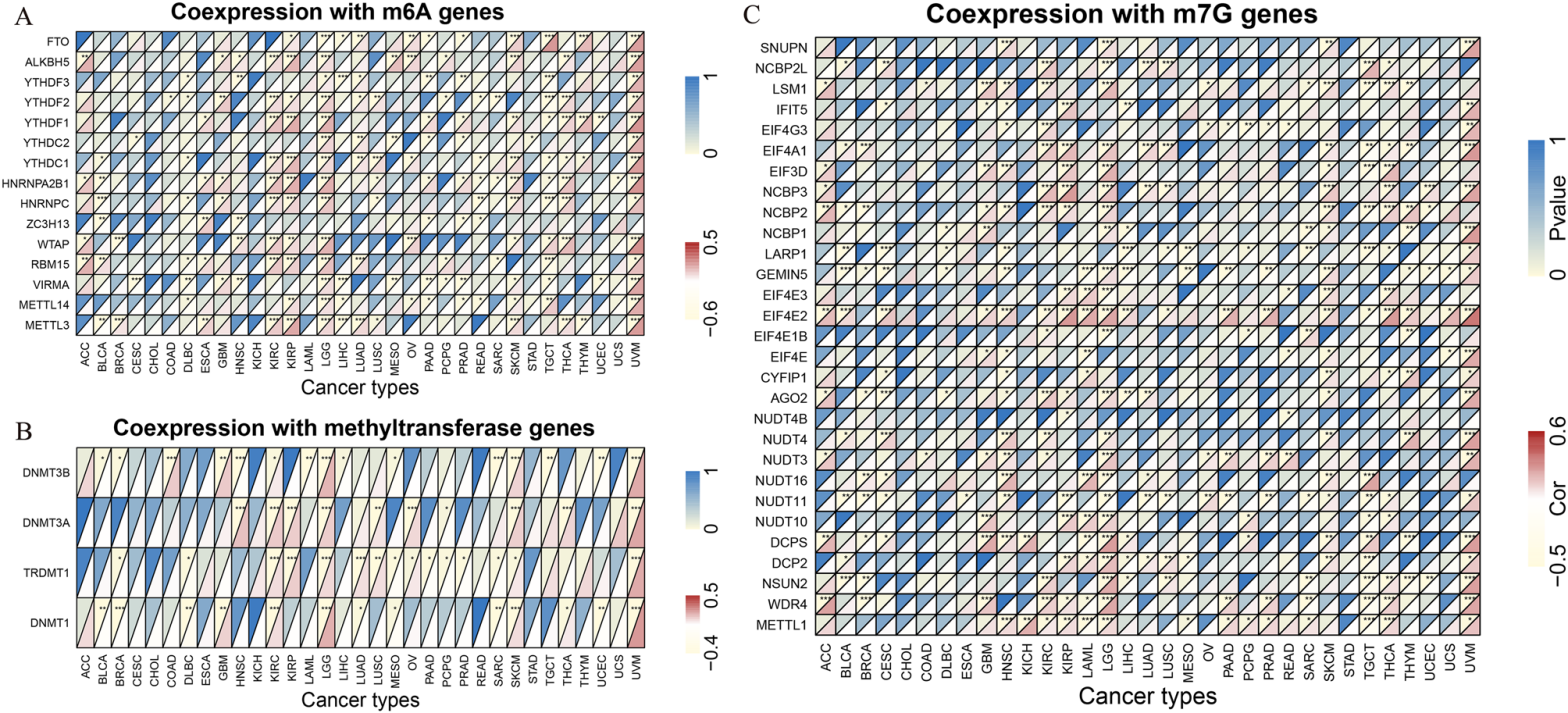
Relationship between RARA-AS1 expression and methylation-related genes. Correlation between RARA-AS1 expression and (**A**) m6A-related genes, (**B**) methyltransferase genes, and (**C**) m7G-related genes. *p < 0.05, **p < 0.01, ***p < 0.001.

The methylation analysis results were divided into three parts. Firstly, the relationship between m6A-related genes and RARA-AS1 was analyzed, and the results showed that RARA-AS1 was significantly correlated with most genes (greater than 10 genes) in KIRP, LGG, TGCT, and UVM (Fig. 6A). We also found that RARA-AS1 was significantly correlated with all methyltransferase genes (DNMT3B, DNMT3A, TRDMT1, and DNMT) in KIRC, LGG, Skin cutaneous melanoma (SKCM), and UVM (Fig. 6B). In addition, m7G genes such as EIF4E2, DCPS, WDR4, and METTL1 were highly correlated with RARA-AS1 in multiple types of cancer (greater than 10 types, Fig. 6C). These relationships between methylation-related genes and RARA-AS1 provide another level of explanation for tumor occurrence and development.

### RARA-AS1 can serve as a biomarker and an independent prognostic factor for glioblastoma

**Figure 7:**
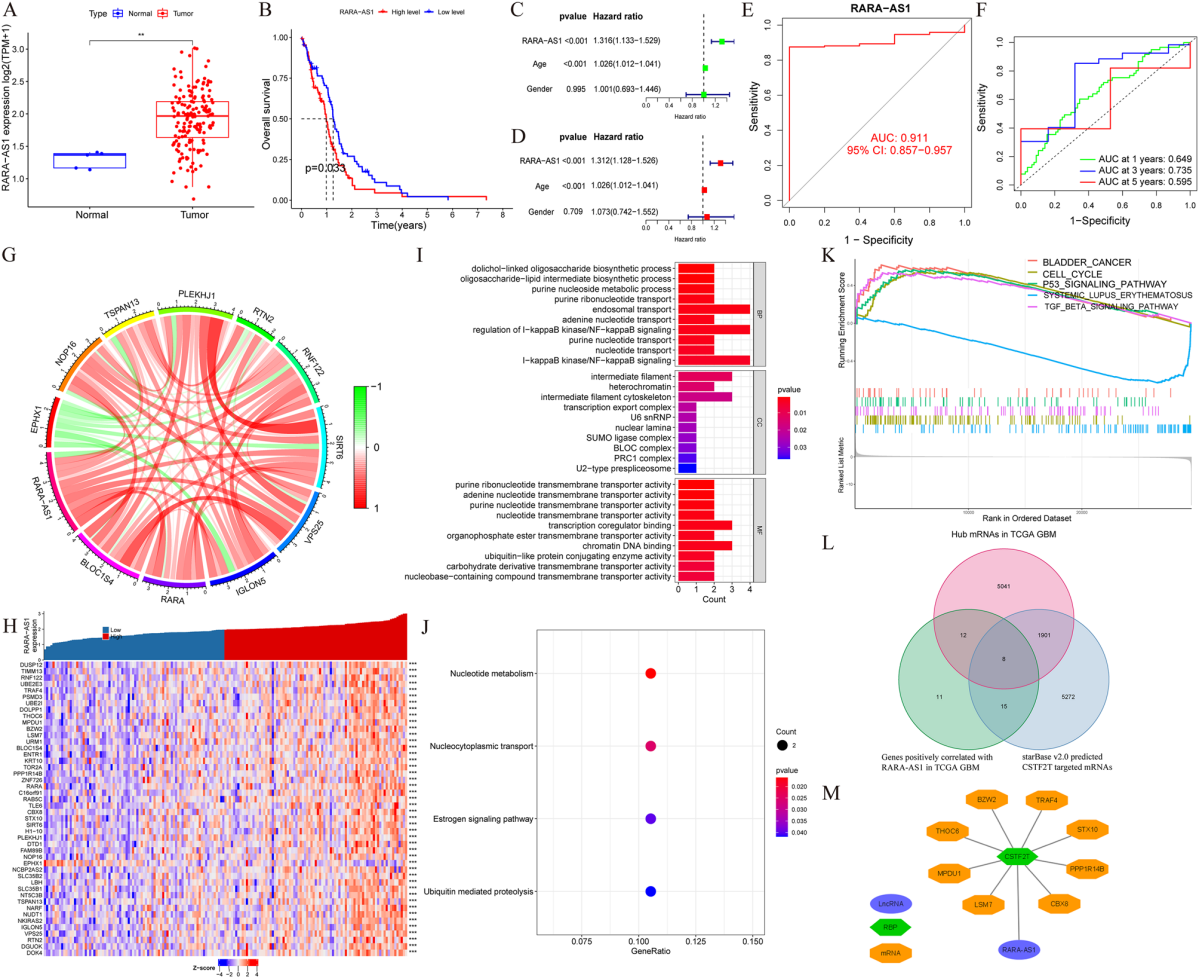
Customized analysis of RARA-AS1 in GBM. (**A**) Boxplot of the RARA-AS1 expression in TCGA; (**B**) Kaplan–Meier survival curve of RARA-AS1 in TCGA; (**C**,**D**) Univariate and multivariate regression analyses of RARA-AS1 in TCGA; (**E**) Diagnostic ROC curve of RARA-AS1in TCGA; (**F**) 1, 3, and 5 year’s prognostic ROC curve of RARA-AS1 in TCGA; (**G**) A circle diagram showing the most related genes to RARA-AS1; (**H**) Heatmap based on differently expressed RARA-AS1 in TCGA; (**I**,**J**) GO and KEGG analyses of RARA-AS1in TCGA; (**K**) GSEA analysis of RARA-AS1 in TCGA; (**L**,**M**) Potential RBP-related mechanisms based on starbase database.

We selected GBM for further analysis based on the following selection criteria: (1) differential expression between cancer and adjacent normal tissues; (2) High-expression RARA-AS1 has a poor prognosis, while low-expression RARA-AS1 has a good prognosis; (3) MSI follows the same trend as OS survival and expression. We found that RARA-AS1 was highly expressed in tumor tissues (Fig. 7A), and the overexpression of this gene was linked to a poor prognosis in patients with GBM (Fig. 7B). In univariate and multivariate Cox regression analyses, RARA-AS1 could serve as an independent prognostic factor for GBM (Fig. 7C,D). ROC curve analysis showed that RARA-AS1 had good diagnostic efficiency (AUC = 0.911, Fig. 7E) and moderate prognostic efficiency. The AUCs for 1-year, 3-year, and 5-year are 0.649, 0.735, and 0.595, respectively (Fig. 7F). Therefore, RARA-AS1 can serve as a biomarker and an independent prognostic factor for glioblastoma.

### Enrichment analysis and potential mechanisms of RARA-AS1 in glioblastoma

To investigate the function of RARA-AS1, we conducted a series of analyses, including gene expression correlation analysis, differential analysis, GO analysis, and KEGG analysis. Genes highly correlated with RARA-AS1 are shown in Fig. 7G. The differential analysis results grouped by RARA-AS1 expression level are displayed in a heatmap in Fig. 7H. Subsequent GO analysis results showed that RARA-AS1 may participate in dolichol-linked oligosaccharide biosynthetic process, intermediate filament, and purine ribonucleotide transmembrane transporter activity (Fig. 7I). KEGG analysis results indicated that RARA-AS1 may participate in pathways such as nucleotide metabolism, nucleocytoplasmic transport, estrogen signaling pathway, and ubiquitin-mediated proteolysis (Fig. 7J). Additionally, GSEA results based on different principles showed that highly expressed RARA-AS1 may participate in pathways such as cell cycle and P53 signaling pathway (Fig. 7K). These functional analyses and pathway predictions provide insights into the mechanism of RARA-AS1.

Based on expression matrix of TCGA GBM, we firstly identified differentially expressed mRNAs and certain mRNAs which had a positive correlation with RARA-AS1. Then the mRNAs which targeted by CSTF2T (RBP, which targeted RARA-AS1) were filtered out using starBase database. Figure 7L shows the final mRNAs targeted by RBP identified by Venn diagram. The RBP-related axis is the RARA-AS1/CSTF2T (RBP)/mRNAs axis, which was used to find mRNAs targeted by RBP. Finally, Cytoscape 3.6.1 software was used to identify and visualize the potential RARA-AS1/CSTF2T (RBP)/mRNAs network, as shown in Fig. 7M.

### Prediction of drug treatment response

**Figure 8:**
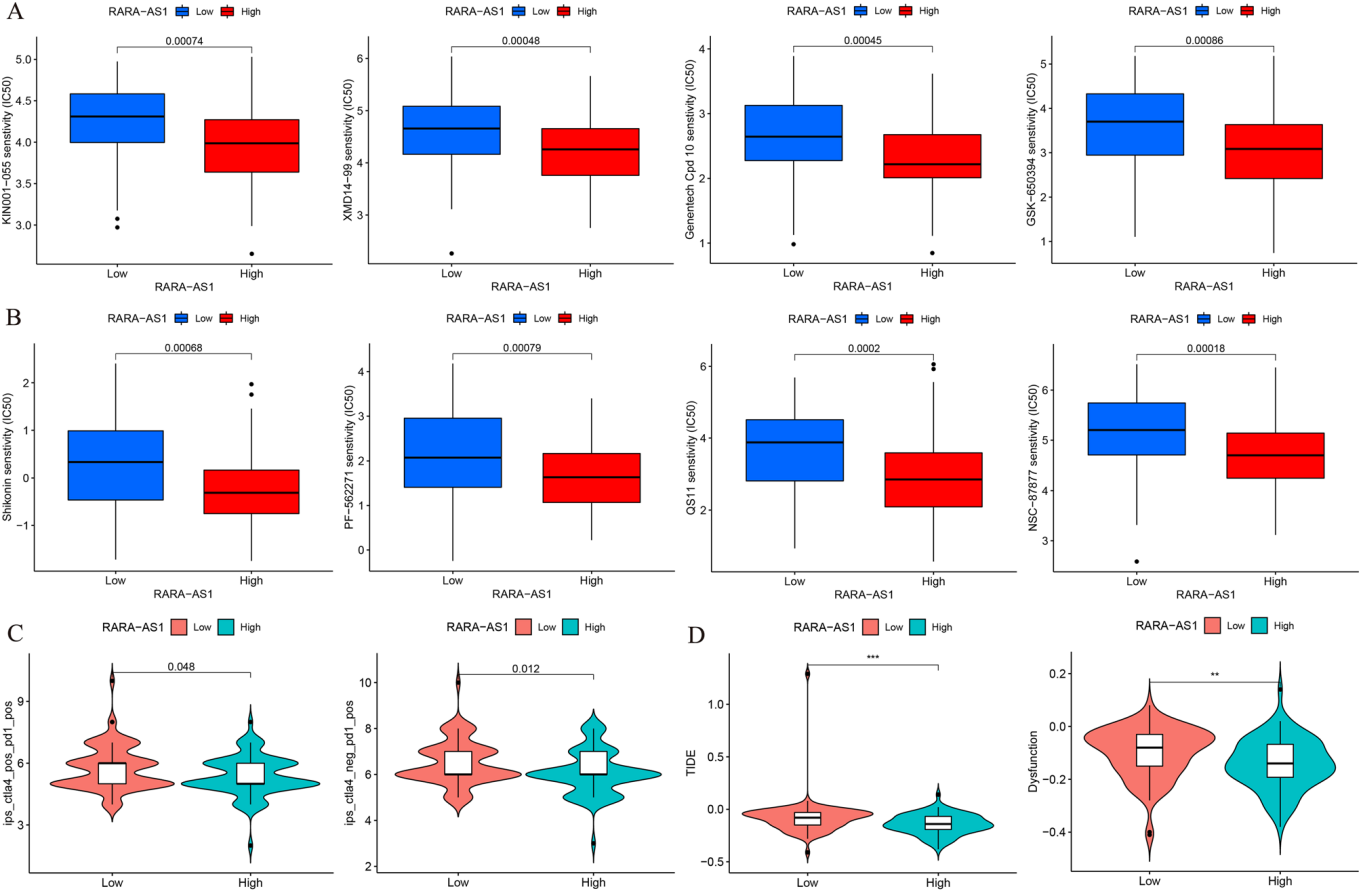
Prediction of responses to various therapy in GBM patients. (**A**,**B**) The different IC50 values in high- and low-expressed RARA-AS1 subgroups; (**C**) Distribution of RARA-AS1 expression in CTLA4 and PD1 scores by TCIA database; (**D**) Distribution of RARA-AS1 expression in TIDE and dysfunction scores by TIDE database.

Based on the R package “pRRophetic”, we predicted drugs in glioblastoma patients and found that compared with the RARA-AS1 low-expression group, drugs such as KIN001-055, GSK-650394, Shikonin, and QS11 had lower IC50 values in the RARA-AS1 high-expression group, indicating that a relatively lower drug concentration can achieve better therapeutic effects in the RARA-AS1 high-expression group (Fig. 8A,B). In addition, data from TCIA showed a significant difference between the RARA-AS1 high and low expression groups in CTLA4 and PD1 co-positive or CTLA4-negative PD1-positive treatments, with statistical significance (Fig. 8C). Meanwhile, TIDE analysis results showed that the RARA-AS1 high-expression group had lower TIDE and Dysfunction scores, which means a lower possibility of immune surveillance escape and higher success rate of immunotherapy than the RARA-AS1 low-expression group (Fig. 8D). As a result, patients who express high levels of RARA-AS1 have a lower survival rate, but have a variety of therapeutic options.

### RARA-AS1 is closely related to proliferation, invasion, and migration of glioblastoma cells

**Figure 9:**
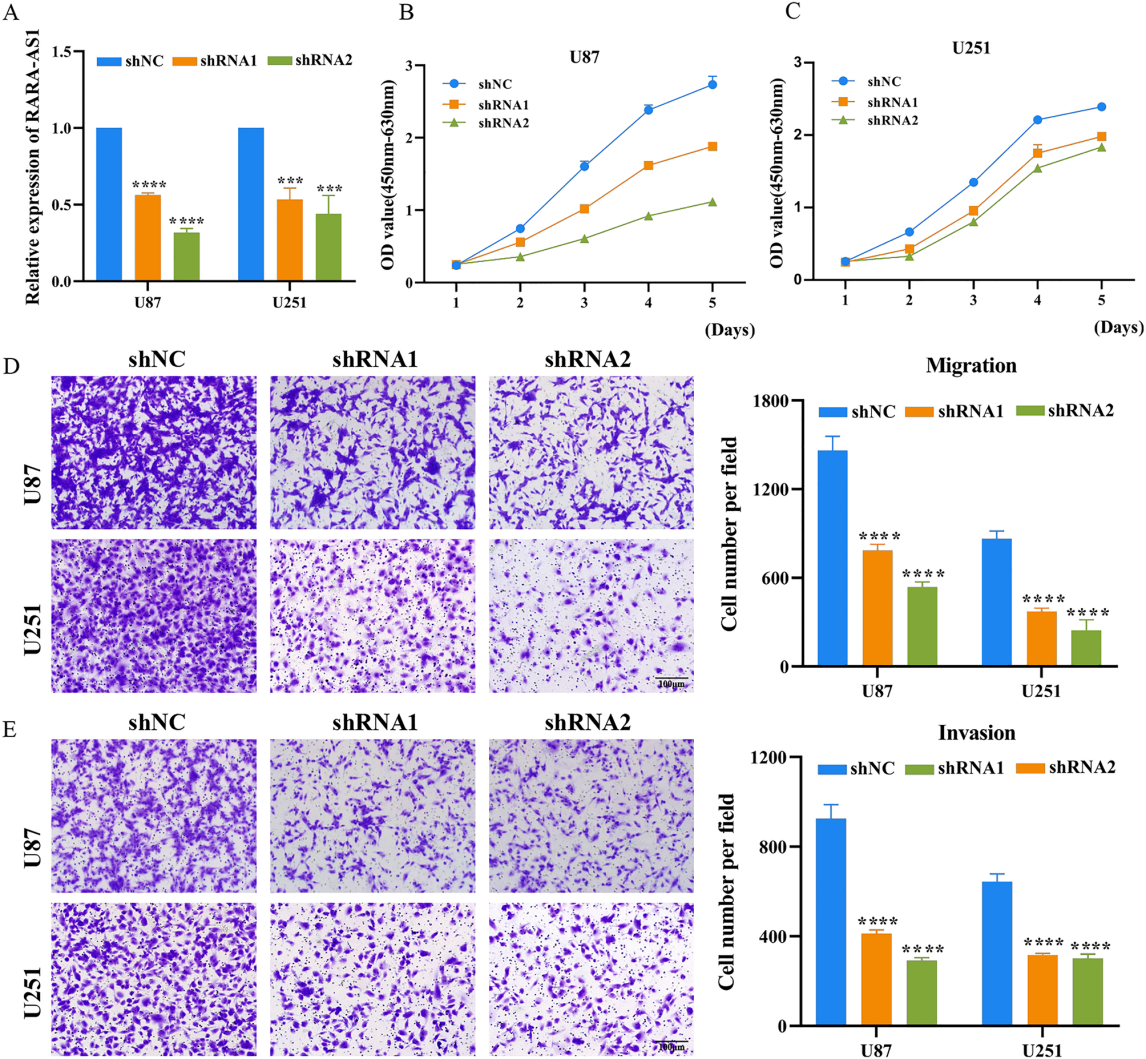
Knockdown of RARA-AS1 expression inhibits cell proliferation and migration of GBM cells. (**A**) Knockdown efficiency of shRNA1 and shRNA2 evaluated by qRT-PCR in GBM cell lines; (**B**) The proliferation of GBM cells was examined by CCK-8 assay; (**C**,**D**) The migration and invasion of GBM cells was examined by transwell assays.

We used two shRNAs (shRNA1 and shRNA2) to knockdown RARA-AS1 expression in U87 and U251 cell lines to observe changes in the biological functions of glioblastoma cells. Figure 9A shows the knockdown efficiency that shRNA1 and shRNA2 subgroups had significant decreases in both cell lines, in which shRNA2 subgroup had a higher efficiency. In the CCK-8 experiment of U87 cells, both shRNA1 and shRNA2 groups showed significantly slower growth rates with the increase of days, compared to the control group, and both could inhibit the proliferation of U87 cells. Especially shRNA2 showed the best inhibitory effect, which is consistent to the trend of the knockdown efficiency (Fig. 9B). The results in U251 were obviously consistent with those in U87 (Fig. 9C). Figure 9D shows that both knockdown groups can inhibit migration ability in both cell lines. The right side shows the quantitative results, where we can clearly and intuitively observe the difference in cell quantity and knockdown effect. In addition, in the invasion experiment conducted simultaneously, the inhibitory ability of both shRNA1 and shRNA2 groups remained stable and consistent with previous results (Fig. 9E).

## Discussion

RARA-AS1 is significantly upregulated in tumor tissues of CHOL, ESCA, GBM, KIRP, LIHC, and THCA. COX regression analysis of OS showed that this gene is a risk factor in GBM, LGG, and UVM, while in DSS's COX analysis, it was a risk factor in COAD, GBM, KIRC, LGG, STAD, and UVM. Therefore, RARA-AS1 is highly expressed in tumor tissues of GBM, and its high expression is associated with poor OS and DSS prognosis of patients, indicating consistent trends in expression and survival. On the other hand, RARA-AS1 is significantly downregulated in tumor tissues of BLCA, CESC, KICH, LUAD, LUSC, and UCEC. In KICH, KIRP, SARC, THYM, and UCS, low expression of RARA-AS1 has been found to be associated with better OS. Therefore, in KICH, the expression and survival trend of RARA-AS1 are consistent. In previous studies, differentially expressed lncRNAs, including RARA-AS1, and their potential targets were successfully identified through blood transcriptome sequencing data. These genes are closely related to immune cell dysregulation in sepsis and may become new biomarkers and therapeutic targets for sepsis^15^. Another study focused more on pediatric sepsis, where 15 key genes, including RARA-AS1, were successfully identified and can serve as diagnostic markers^26^. However, in the field of tumors, this study is the first to investigate the expression of RARA-AS1 at the RNA level and its relationship with survival. Further validation from the cellular, tissue, and serum levels is needed to explore its differential expression and whether it can become a key biomarker.

DNA mismatch repair deficiencies in tumor tissue cause MSI. This phenomenon is also an important clinical tumor marker. Patients with more MSI phenomena have a better prognosis than those with fewer MSI phenomena because T lymphocytes are activated to recognize new antigens^27^. Therefore, patients with GBM, CHOL, and ACC will have poor prognosis with RARA-AS1 high expression and less MSI phenomena. This is consistent with our previous analysis that GBM patients with high expression of RARA-AS1 have poor prognosis, which is why we focus on the analysis of GBM. TMB is highly correlated with the efficacy of PD-1/PD-L1 inhibitors^28^. Unfortunately, in the correlation analysis between RARA-AS1 and TMB, only BRCA showed some association. However, in the subsequent analysis of drug response prediction in GBM, significant differences were observed in PD1 and CTLA4 treatment between high and low expression groups of RARA-AS1. Therefore, further research at the cell and animal levels is needed to explore whether specific PD1/PD-L1-related treatments are effective for different GBM patients with different RARA-AS1 expression.

The tumor microenvironment is a complex system, generally composed of blood vessels, tumor cells, immune cells, inflammatory cells, and fibroblasts. The tumor immune microenvironment (TIME) is an important part of tumor immunity, and it is often composed of various immune cells such as macrophages, neutrophils, and dendritic cells^29^. lncRNAs have different cellular functions in TIME^30^. Based on our research results, we have made some bold speculations, such as RARA-AS1-induced T cells follicular helper cells in GBM to participate in immune responses, and RARA-AS1-promoted Macrophages M2 infiltration in LUAD to participate in immune responses. In previous studies, correlation analysis was also applied, and the gene JMJD8 was found to be highly correlated with M2 macrophages, confirming its potential as a therapeutic target^31^.

In addition, RNA methylation is also an important component of epigenetic modification and is closely related to the occurrence, development, and prognosis of tumors. Among them, m6A and m7G are common forms. Classic methyltransferases are also an indispensable part of methylation-related analysis. METTL3, an m6A methyltransferase, has oncogenic function in several human cancer^32^. By reducing METTL3 expression, cancer cells can be less capable of migrating, invading, and undergoing epithelial-mesenchymal transitions^33,34^. YTHDF1 is highly expressed in colon cancer and knocking it down significantly inhibits the tumorigenicity of CRC cells in vitro^35^. Our research found that RARA-AS1 is significantly correlated with various m6A and m7G-related methyltransferases and methyltransferases in various tumors. RARA-AS1 is likely to be associated with these genes, affecting methylation, and thereby affecting tumor occurrence and progression.

In exploring the pathway enrichment of RARA-AS1 in glioblastomas, it was found that the gene may be involved in pathways such as nucleotide metabolism, nucleocytoplasmic transport, cell cycle, and the p53 signaling pathway. Previous studies have reported how lincRNA regulates or affects tumor phenotypes such as proliferation, invasion, and migration through these pathways. For example, Gandhi et al. discovered a lncRNA, lincNMR, which regulates tumor cell proliferation by controlling nucleotide metabolism through the YBX1-RRM2-TYMS-TK1 axis^36^. Additionally, lincRNA SNHG18 has been shown to inhibit nuclear-cytoplasmic transport in glioblastoma cells by directly binding to α-enolase ENO1, thereby promoting glioblastoma cell invasion and migration^37^. Numerous lincRNAs have been shown to participate in related pathways and affect cancer cell biology, including the cell cycle pathway and P53 signaling pathway^38,39^. Based on our research results and previous studies, lincRNA RARA-AS1 is likely to affect proliferation and migration of glioblastoma through the mentioned pathways, warranting further investigation.

This study has nice innovations and strengths. Firstly, this is the first time we focus on the lncRNA RARA-AS1 in tumours, analysing the role played by RARA-AS1 in tumours from a more comprehensive perspective. In addition, based on the results of the pan-cancer analysis, and the results of the GSEA analysis previously performed in GBM, we conducted relevant experiments. This is also the first time to explore: the effect of RARA-AS1 on the biological behaviour of GBM tumours. Finally, we also performed a possible RBP mechanism exploration. Although it is only at the theoretical level and needs experimental verification, it is a bold attempt.

However, there are some limitations to this article that are worth noting and exploring in subsequent research. Firstly, this study was retrospective, and future prospective studies are needed to validate the expression of RARA-AS1 and its correlation with patient prognosis and tumor immunity. Moreover, RARA-AS1’s ability to serve as a biomarker and independent prognostic factor also needs to be validated in large-scale tissue and serum samples. Finally, the exploration of mechanisms in this paper needs to be given more attention. In addition to RBP-related mechanisms, we should draw on a variety of excellent algorithms in the future to further analyze and validate the lincRNA-miRNA mechanisms as well, such as those based on semi-supervised interactome network^40^, graph convolutional neural network and conditional random field^41^, logistic matrix factorization with neighbourhood regularized^42^, as well as the auto-encoder and non-negative matrix factorization^43^. Besides, many theoretical computational models have been applied in the field of regulatory pathways. a Cahn–Hilliard phase-field model paired with Ginzburg–Landau free-energy scheme proposed by Xu et al.^44^ and Mathematical modelling combined with experimental analysis of NLRP1b inflammasome signalling by Li et al.^45^ are both good examples of the use of theoretical computational models. Last but not least, deep machine learning will also be our way forward in the future. Wang et al.^46^ and Sun et al.^47^ were used to predict, develop small molecule drugs and analyze metabolite-disease relationships respectively. In the future, this can be applied to our lncRNAs, not only to predict upstream mechanisms, but also to analyse downstream metabolites and even to develop new drugs based on these algorithms. This is indeed our shortcoming and at the same time the direction of our endeavour.

Overall, our study found that RARA-AS1 is aberrantly expressed in different cancers. Abnormal expression of RARA-AS1 is associated with clinical staging, prognosis, MSI, TMB, RNA methylation, and the tumor immune microenvironment in pan-cancer. Therefore, RARA-AS1 may prove to be a potential prognostic biomarker for different types of cancer, affecting prognosis and immune therapy. It is also an independent risk factor affecting prognosis in GBM and an important factor affecting proliferation and migration in GBM.

### Supplementary Information

Supplementary Figure 1.

Supplementary Legends.

## Data Availability

The RNA-sequencing data and corresponding clinical information were downloaded from the Cancer Genome Atlas (TCGA) database (https://portal.gdc.cancer.gov/), via selecting the corresponding categories for the different tumors, including “transcriptome profiling” and “clinical”, respectively. The version of genome file was GRCh38.p13.
