# Supplementary figures and images for: LncRNA RARA-AS1 could serve as a novel prognostic biomarker in pan-cancer and promote proliferation and migration in glioblastoma
Source: Sci Rep. 2023 Oct 13;13:17376. doi: 10.1038/s41598-023-44677-4 (PMC10575974; doi:10.1038/s41598-023-44677-4)

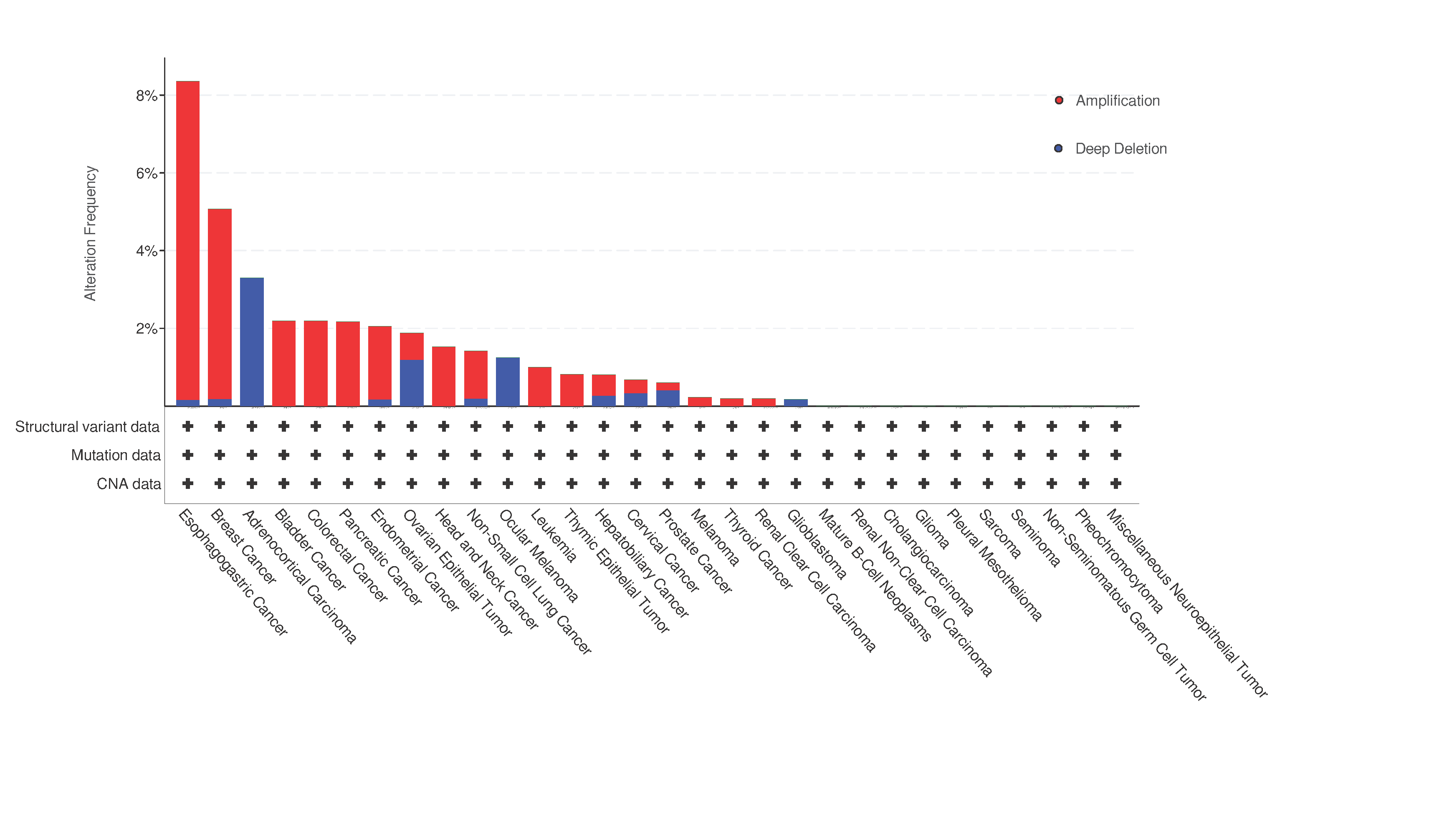

Supplement: Supplementary file 1 — Supplementary Figure 1. [file 41598_2023_44677_MOESM1_ESM.tif]
